# Supplementary material for: Small Angle X-ray Scattering, Molecular Modeling, and Chemometric Studies from a Thrombin-Like (Lmr-47) Enzyme of Lachesis m. rhombeata Venom
Source: Molecules. 2021 Jun 28;26(13):3930. doi: 10.3390/molecules26133930 (PMC8271572; doi:10.3390/molecules26133930)
Supplement: Supplementary file 1 [file molecules-26-03930-s001.zip › molecules-1183063-supplementary.pdf]

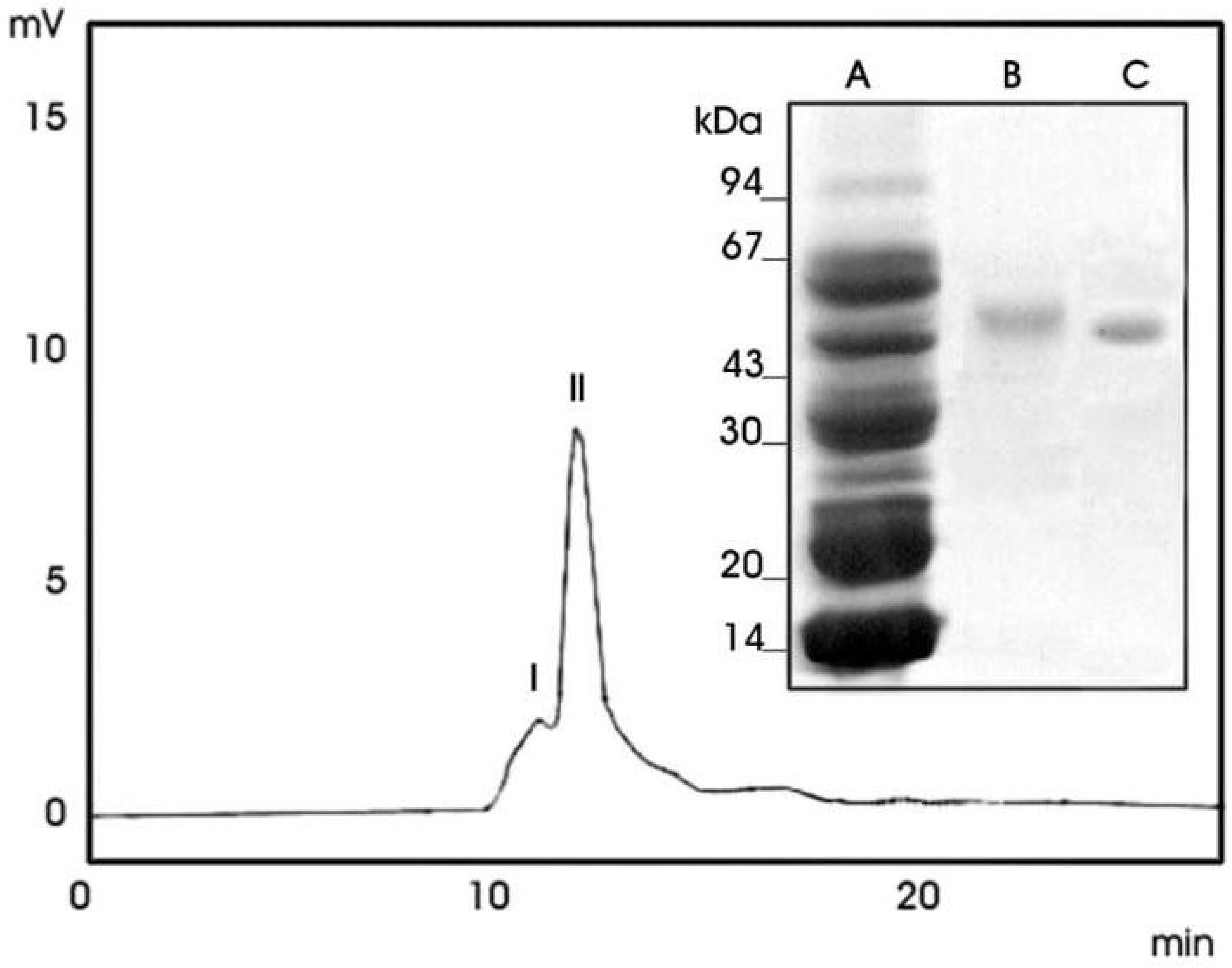
mV
15
10
5
0
I
II
0
10
20
min
A
B
C
kDa
94
67
43
30
20
14

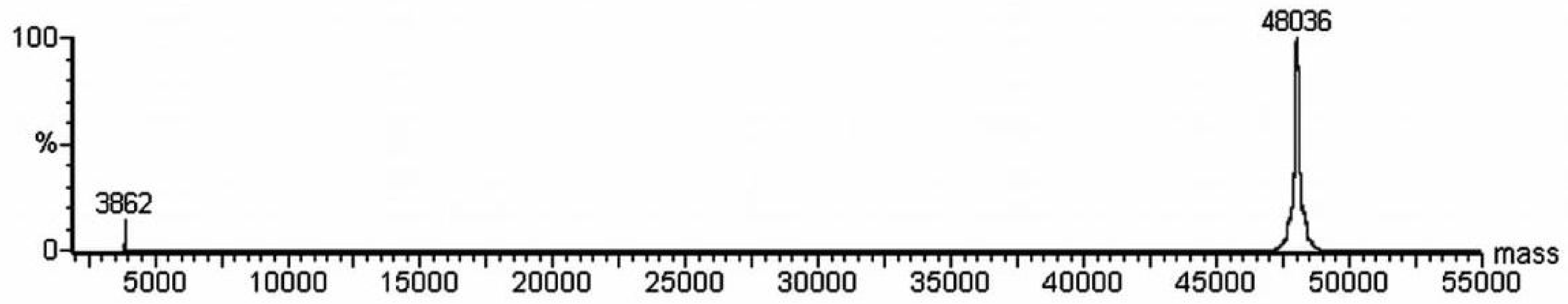

48036
100
%
3862
0
mass
5000
10000
15000
20000
25000
30000
35000
40000
45000
50000
55000

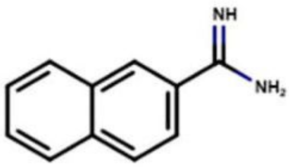
1

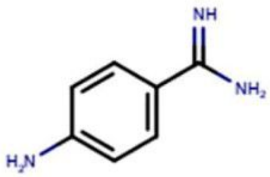
2

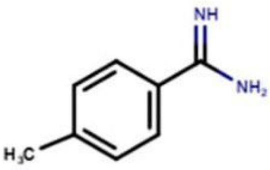
3

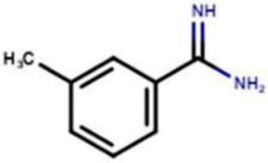
4

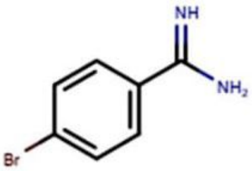
5

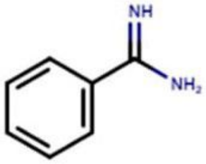
6

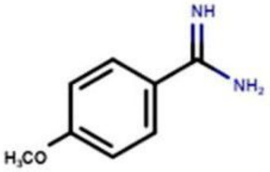
7

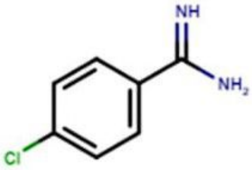
8

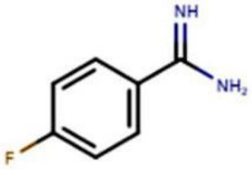
9

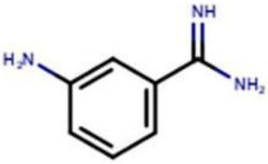
10

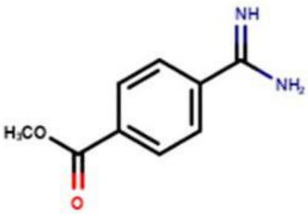
11

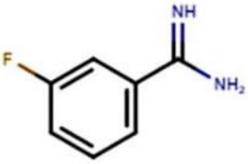
12

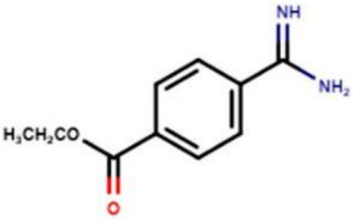
13

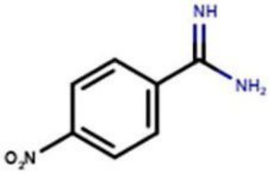
14

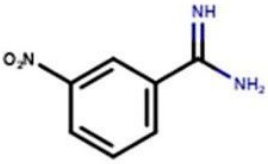
15

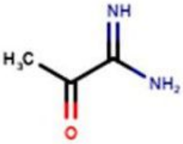
16

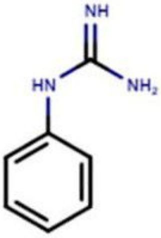
17

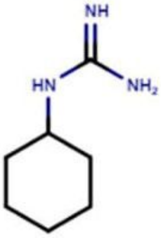
18

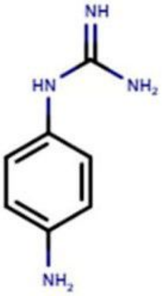
19

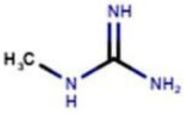
20

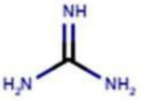
21

Supplementary Table 1

Inhibitor	M.Vol. /Å^3	E_{HOMO}[a] /eV	E_{LUMO}[a] /eV	N-H[a] /Å	Q[a]	□[a]/D	ΔH[a] /kcalmol^{-1}
1	126.00	8.784	-0.399	0.998	-0.0287	2.776	60.9720
2	88.00	8.729	0.137	0.994	0.0794	3.450	40.5330
3	117.00	9.580	0.256	0.997	-0.0739	2.946	35.1098
4	105.00	9.580	0.026	1.119	-0.0739	2.946	35.1098
5	100.00	9.735	-0.435	0.997	-0.1567	1.808	47.9454
6	84.00	9.687	-0.005	0.997	-0.1166	2.791	42.7341
7	119.00	9.206	-0.034	0.997	0.0928	3.183	4.5625
8	94.00	9.695	-0.350	0.997	-0.0504	1.892	35.8670
9	92.00	9.688	-0.347	0.997	0.1038	1.729	-2.3107
10	110.00	8.768	0.096	0.997	0.0055	2.820	41.1608
11	103.00	10.131	-0.953	0.997	-0.1424	4.092	-40.6402
12	92.00	9.688	-0.347	0.997	-0.1724	1.729	-2.3107
13	127.00	9.371	0.068	0.997	-0.0546	3.175	-45.0090
14	104.00	10.289	-1.412	0.997	-0.1210	3.573	47.4221
15	122.00	10.199	-1.302	.0997	-0.1369	4.861	47.5130
16	72.00	10.112	0.191	0.996	0.2810	1.687	-22.1760
17	169.00	8.658	0.287	0.996	-0.2737	2.369	48.8128
18	138.00	9.246	1.365	0.996	-0.3080	1.717	-5.2640
19	157.00	7.943	0.295	1.100	-0.2559	3.006	48.2390
20	64.00	9.245	1.337	1.001	-0.3057	1.699	18.9915
21	63.00	9.731	1.413	0.996	-0.3458	2.160	18.6993

[a]Calculated with AM1, MOPAC v7.0 package
